# Enamel Matrix Derivative Suppresses Chemokine Expression in Oral Epithelial Cells

**DOI:** 10.3390/ijms241813991

**Published:** 2023-09-12

**Authors:** Layla Panahipour, Sara Botta, Azarakhsh Oladzad Abbasabadi, Zohreh Afradi, Reinhard Gruber

**Affiliations:** 1 Department of Oral Biology, University Clinic of Dentistry, Medical University of Vienna, Sensengasse 2a, 1090 Vienna, Austria; layla.panahipour@meduniwien.ac.at (L.P.); sslmit.sara1995@gmail.com (S.B.); az.azar66@gmail.com (A.O.A.); zohreh_afradi@yahoo.com (Z.A.); 2 Department of Periodontology, School of Dental Medicine, University of Bern, 3010 Bern, Switzerland; 3 Austrian Cluster for Tissue Regeneration, 1200 Vienna, Austria

**Keywords:** enamel matrix derivative, oral epithelial cells, chemokines, bioassay, inflammation, periodontitis

## Abstract

Epithelial cells in periodontitis patients increasingly express chemokines, suggesting their active involvement in the inflammatory process. Enamel matrix derivative (EMD) is an extract of porcine fetal tooth germs clinically applied to support the regrowth of periodontal tissues. Periodontal regeneration might benefit from the potential anti-inflammatory activity of EMD for epithelial cells. Our aim was, therefore, to set up a bioassay where chemokine expression is initiated in the HSC2 oral squamous carcinoma cell line and then test EMD for its capacity to lower the inflammatory response. To establish the bioassay, HSC2 cells being exposed to TNFα and LPS from *E. coli* (*Escherichia coli*) or *P. gingivalis* (*Porphyromonas gingivalis*) were subjected to RNAseq. Here, TNFα but not LPS caused a robust increase of chemokines, including CXCL1, CXCL2, CXCL8, CCL5, and CCL20 in HSC2 cells. Polymerase chain reaction confirmed the increased expression of the respective chemokines in cells exposed to TNFα and IL-1β. Under these conditions, EMD reduced the expression of all chemokines at the transcriptional level and CXCL8 by immunoassay. The TGF-β receptor type I kinase-inhibitor SB431542 reversed the anti-inflammatory activity. Moreover, EMD-activated TGF-β-canonical signaling was visualized by phosphorylation of smad3 and nuclear translocation of smad2/3 in HSC2 cells and blocked by SB431542. This observation was confirmed with primary oral epithelial cells where EMD significantly lowered the SB431542-dependent expression of CXCL8. In summary, our findings suggest that TGF-β signaling mediates the effects of EMD to lower the forced expression of chemokines in oral epithelial cells.

## 1. Introduction

Periodontal health is difficult to maintain as the periodontium must steadily defend against the microbial and other burdens inherent to the oral cavity [1,2,3]. There is also mechanical damage to the gingiva coming from exaturated mastication that the periodontium has to deal with [4], however, physiological masticatory loaging is required for periodontal homeostasis [5]. Thus, the gingiva and the junctional epithelium with the periodontal ligament underneath build a vital barrier toward the oral cavity equipped with a defense system driven by inflammation [6,7,8,9]. There is consequently a delicate balance of the immunological defense to maintain periodontal homeostasis and the possible shift towards an overshooting and chronic inflammation. This catabolic scenery is mainly driven by biofilms that resist the immunological defense system, culminating in the loss of periodontal structures and ultimately leading to tooth loss [10,11,12,13,14,15]. Understanding the pathological mechanisms causing periodontitis at a molecular and cellular level is the fundament of a successful therapy.

Periodontal regeneration requires removing biofilms and other sources causing chronic inflammation [16,17,18,19]. Only then can a series of clinical approaches be applied with the overall goal of regenerating the periodontal tissues that were deteriorated by the catabolic inflammatory environment. Traditionally, neutrophils, macrophages, and lymphocytes have been blamed for being responsible for causing this inflammatory environment. It was, however, not until recently that a sophisticated single-cell RNA sequencing approach identified epithelial cells to promote leucocyte recruitment [20]. Epithelial cells in periodontitis increasingly express chemokines, including chemokine (C-X-C motif) ligand 1 (CXCL1), critical for recruiting neutrophils [20,21], and it is the neutrophil extracellular traps and histones triggering the early catabolic environment of periodontitis [22]. Oral epithelial cells damaged by masticatory forces may contribute to the inflammation of the periodontium [4]. Under the premise that the oral epithelium contributes to the overall inflammatory defense by the release of chemokines, there is a rational to study the modulation of chemokine expression by established as well as future periodontal therapies.

Enamel matrix derivative (EMD), an extract of porcine fetal tooth germs [23,24,25,26], is clinically applied to support the regrowth of periodontal tissues, and numerous clinical studies have tested its efficacy [27,28,29,30]. Even though the underlying molecular and cellular mechanisms are not fully understood, the effects of EMD might involve the modulation of local inflammation [31]. This has prompted us to study the impact of EMD on macrophages in an LPS-induced inflammatory environment [32,33]. To this aim, EMD significantly attenuated the LPS-induced expression of lead cytokines in RAW 264.7 and primary macrophages, an activity that was attributed to TGF-β, a pleiotropic growth factor found in EMD [32,33]. The TGF-β activity was further attributed to mediating the effects of EMD on osteoclastogenesis [34], adipogenesis [35], and changes in the genetic signature of gingival fibroblasts [36]. Moreover, TGF-β was hypothetically proposed to mediate EMD effects on gene expression changes in HSC2 oral squamous cell carcinoma epithelial-like cells [37]. Thus, the question arises if EMD and its TGF-β activity can lower the forced expression of chemokines in HSC2 cells. 

To answer this question, we have aimed to establish a bioassay to simulate chemokine expression by oral epithelial cells in periodontitis [20]. Among the chemokines identified in vivo, our RNAseq identified CXCL1 and a series of other chemokines to be strongly upregulated in HSC2 oral epithelial cells exposed to TNFα but not to LPS from either *E. coli* or *P. gingivalis*—serving as a basis for the bioassay. The bioassays allowed us to generate data suggesting that EMD can lower the cytokine-induced expression of chemokines and that the effects can be attributed to its TGF-β activity.

## 2. Result

### 2.1. TNFα but Not LPS Increases Chemokine Expression in HSC2 Cells

**Figure 1:**
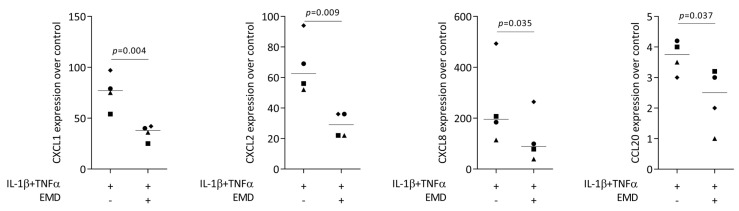
Gene expression of chemokines CXCL1, CXCL2, CXCL8, and CCL20 in HSC2 cells under TNFα and IL-1β stimulation. A total of 300 µg/mL EMD significantly reduced chemokine expression. Data are expressed as x-fold over the respective untreated controls. Data points represent four independent experiments. Statistical analysis was based on paired *t*-tests, and *p*-values are indicated.

**Figure 2:**
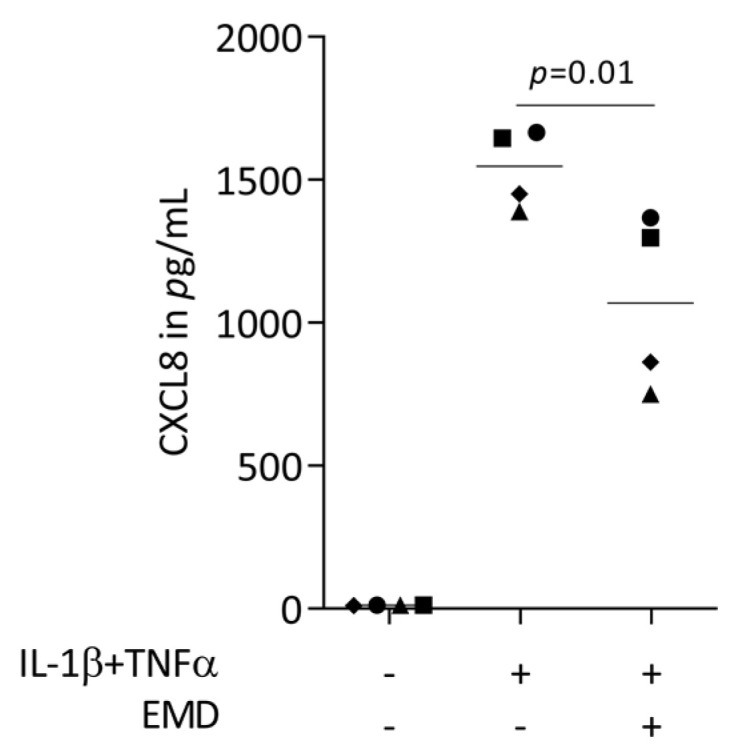
CXCL8 protein secretion in HSC2 cells under TNFα and IL-1β stimulation. 300 µg/mL EMD significantly reduced protein secretion. Data points represent four independent experiments. Statistical analysis was based on RM one-way ANOVA, with the Geisser–Greenhouse correction tests and *p*-values are indicated.

First, we performed a screening for chemokines being expressed in HSC2 cells exposed to TNFα and LPS from *E. coli* or *P. gingivalis*. RNAseq revealed that TNFα but none of the two LPS sources caused a robust increase of chemokines, including CXCL1-3, CXCL8-11, CXCL16, CCL5, and CCL20 (Supplementary Table S1). In our RT-PCR-based bioassay with HSC2 cells exposed to TNFα and IL-1β, we could see a robust increase of CXCL1, CXCL2, CXCL8, and CCL20 expression (Figure 1) but also of CXCL10, CXCL11, CXCL12, and CCL5. When HSC2 cells were simultaneously exposed to EMD, the forced expression of CXCL1, CXCL2, CXCL8, and CCL20 was significantly reduced (Figure 1). These findings were supported by an immunoassay showing CXCL8 reduction by EMD (Figure 2). However, EMD failed to significantly lower CXCL10, CXCL11, CXCL12, and CCL5 expression. These findings suggest, first, that the HSC2-based bioassay is a model to study the expression changes of chemokines and, second, that EMD can reduce a selected panel of chemokines.

### 2.2. EMD Causes Phosphorylation of smad3 and Translocation of smad2/3 in HSC2 Cells

**Figure 3:**
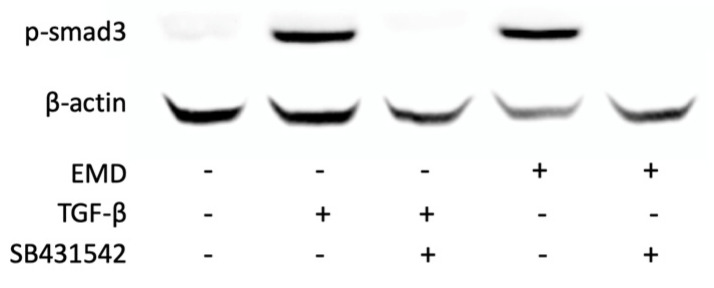
Smad3 phosphorylation in HSC2 cells. Both, 300 µg/mL EMD and 10 ng/mL TGF-β induced the phosphorylation of smad3. This effect was abolished by the TGF-β receptor type I kinase-inhibitor SB431542.

**Figure 4:**
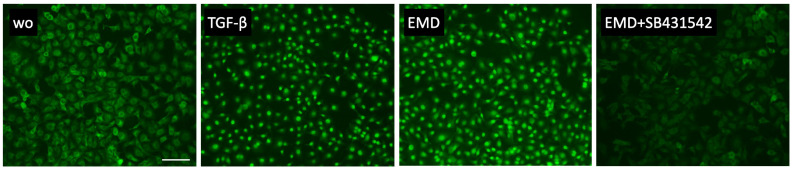
Smad2/3 nuclear translocation in HSC2 cells. Both, 300 µg/mL EMD and 10 ng/mL TGF-β induced the nuclear translocation of smad2/3 indicated by the immunofluorescence staining. This effect was reversed with the application of the TGF-β receptor type I kinase-inhibitor SB431542. The scale bar represents 100 µm.

Based on a series of previous experiments indicating TGF-β activity of EMD [32,33,34,35,36], we tested the activation of canonical TGF-β signaling in HSC2 cells. In support of this assumption, EMD induced the phosphorylation of smad3 (Figure 3) and the nuclear translocation of smad2/3 (Figure 4). Moreover, in the presence of the TGF-β receptor type I kinase-inhibitor SB431542, EMD failed to induce the phosphorylation of smad3 (Figure 3) and the nuclear translocation of smad2/3 (Figure 4). Taken together these data not only confirm that EMD activates canonical TGF-β signaling in HSC2 cells; the data further supports the responsiveness of the HSC2 bioassay for TGF-β. 

### 2.3. Blocking the TGF-β Signaling Reverses the Ability of EMD to Reduce CXCL8 Expression in HSC2 Cells

**Figure 5:**
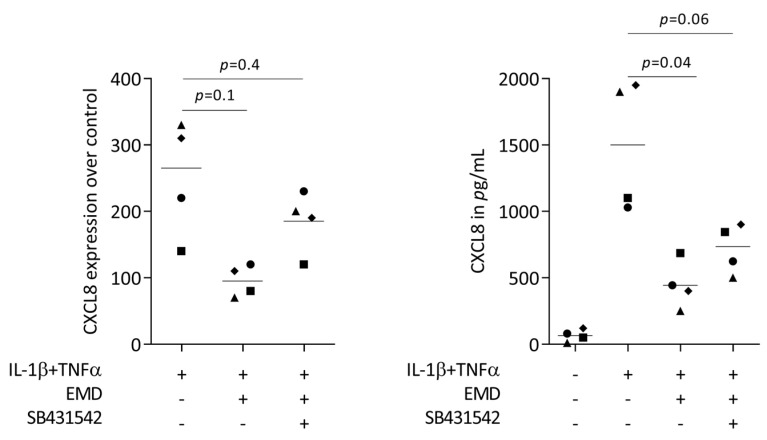
SB431542 partially reverses the activity of EMD on CXCL8 expression. HSC2 cells under IL-1β and TNFα stimulation were exposed to 300 µg/mL EMD in the absence and presence of the TGF-β receptor type I kinase-inhibitor SB431542. Data points represent four independent experiments. Statistical analysis was based on RM one-way ANOVA, with the Geisser–Greenhouse correction tests and *p*-values are indicated.

To understand if canonical TGF-β signaling is necessary for EMD to reduce the chemokine expression of HSC2 cells, SB431542 was implemented in the bioassay. In support of previous observations [32,34,35,36], SB431542 partially reversed the ability of EMD to lower the forced expression of CXCL8 in HSC2 cells (Figure 5). Moreover, recombinant TGF-β reduced the chemokine expression of HSC2 cells (Supplementary Table S2). However, the ability of EMD to lower the expression of chemokines was not associated with diminished phosphorylation of p38 and p65 (Supplementary Figure S1). These data indicate that the TGF-β activity is at least partially required to mediate the effects of EMD in lowering the CXCL8 expression in HSC2 cells.

### 2.4. Primary Oral Epithelial Cells Are Comparable with HSC2 Cells with Respect to CXCL8 Expression

**Figure 6:**
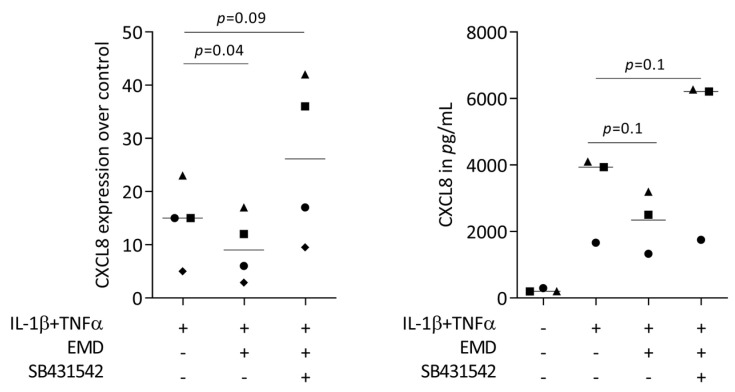
SB431542 partially reverses the activity of EMD on CXCL8 expression. Primary oral epithelial cells under IL-1β and TNFα stimulation were exposed to 300 µg/mL EMD in the presence and absence of the TGF-β receptor type I kinase-inhibitor SB431542. Data points represent independent experiments. Statistical analysis was based on RM one-way ANOVA, with the Geisser–Greenhouse correction tests and *p*-values indicated.

Considering that HSC2 are oral squamous carcinoma cells and may potentially not represent the nonmalignant situation, we aimed to confirm the findings with primary oral epithelial cells. In this setting as well, TNFα and IL-1β provoked primary oral epithelial cells to increasingly express CXCL8, however weaker than the HSC2 cells (Figure 6). Moreover, exposure of the primary oral epithelial cells to EMD significantly reduced the forced CXCL8 expression even though the immunoassay only showed a trend towards CXCL8 reduction (Figure 6). Interestingly, there was a trend that SB431542 even increased CXCL8 expression above the TNFα and IL-1β control levels. These data indicate that HSC2 cells at least partially reflect the behavior of primary oral epithelial cells for testing the impact of EMD on chemokine expression. 

## 3. Discussion

Oral epithelial cells of the mucosa are more than a passive barrier shielding away the underlying connective tissue from the pleiotropy triggers accumulating in the oral cavity and the damage caused by mastication [1]. Oral epithelial cells can become a source of chemokines and cytokines driving local immunity to mediate host defense during homeostasis; however, immunity can shift towards tissue destruction when chronic inflammation dominates the scene [4,20]. Thus, oral epithelial cells have received increasing attention for their active participation in modulating the local inflammatory environment by their release of chemokines, and it mainly the chemokines controlling the immigrating of neutrophils, antigen-presenting cells, and lymphocytes into an inflamed tissue [4,20]. Considering this scenario, we have screened for chemokines being expressed in HSC2 oral epithelial cells when exposed to TNFα and LPS from *E. coli* and *P. gingivalis*. One major finding was that HSC2 epithelial cells increasingly express CXCL1-3, CXCL8-11, CXCL16, CCL5, and CCL20 in response to TNFα exposure. This RNAseq screening approach allowed us to establish a bioassay to test potential modulators of chemokine expression, including EMD. 

EMD, commonly used in the clinic to support periodontal regeneration [27,28,29,30], is among the possible modulators of an inflammatory response. This assumption is based on previous reports that EMD significantly reduced the LPS-induced cytokine expression in macrophages [32,33] and cytokine-induced expression of IL6 in mouse ST2 bone marrow stromal cells [32]. Consistent with previous reports, we report here that EMD was capable of reducing the cytokine-induced chemokine expression in the HSC2 cells that were most obvious for CXCL1, CXCL2, CXCL8, and CCL20. However, there was also a trend that EMD lowers the induced expression of the other chemokines, namely CXCL10, CXCL11, CXCL12, and CCL5. We have thus concluded that HSC2 cells are a vital bioassay to study the expression of chemokines by a simulated inflammatory environment and take the advantage of this setting for testing the modulation of chemokine expression by EMD. Yet, HSC2 cells are immortal as they originate from an oral squamous cell carcinoma, and we had to confirm their basic response with primary oral epithelial cells. Consistently, EMD lowered the cytokine-induced expression of CXCL8 in primary oral epithelial cells. However, we have to admit that the primary oral epithelial cells showed a less robust CXCL8 response to IL-1β and TNFα, and the impact of the TGF-β receptor type I kinase-inhibitor SB431542 was even more pronounced than with HSC2 cells. Hence, the findings obtained with the HSC2 cells should be interpreted carefully with respect to primary cells and even tissues. Considering this premise, HSC2 cells remain a suitable bioassay to study the modulation of cytokine-induced chemokine expression. 

The question arises of what could be the underlying molecular mechanism that makes EMD reduce chemokine expression. The answer might be related to TGF-β that mediates at least part of the EMD activity in vitro, e.g., its anti-inflammatory activity in LPS-induced macrophages [32,33], its suppression of adipogenesis [35], its ability to support osteoclastogenesis [34] and its driving gene expression in oral fibroblasts [36]. In support of previous research with EMD-treated epithelial SCC25 and fibroblastic Gin-1 cells [38], we observed that EMD initiates canonical TGF-β signaling indicated by the phosphorylation and nuclear translocation of smads in HSC2 cells. More evidence comes from our observations that the TGF-β receptor type I kinase-inhibitor SB431542 partially reversed the activity of EMD to lower CXCL8 expression. SB431542 was also reported to block EMD effects in other in vitro studies [32,34,35,36]. Our observations are further supported by others showing that TGF-β-induced smad2/3 blocks the CXCL1 promoter in fibroblasts [39,40], and the expression of breast cancer stromal CXCL1 is inversely correlated with the expression of TGF-β signaling [40]. It is thus reasonable to suggest that the TGF-β activity intrinsic to EMD is at least partially responsible for its lowering activity on chemokine expression.

The clinical interpretation of our RNAseq findings remains at the level of speculation, but considering that in vivo, oral epithelial cells express increasing amounts of CXCL1, CXCL3, CXCL5, CXCL16, CCL20, and CCL28 [20], our in vitro RNAseq analysis identified all chemokines but not CXCL5 being increased under inflammatory conditions. In our RT-PCR analysis, we highlight CXCL1 and CCL20, representing the in vivo situation [20], but also included other chemokines identified by our RNAseq. Surprisingly, epithelial cells in periodontitis patients do not show increasing levels of chemokines such as CXCL8 and other chemokines we have identified in our RNAseq assay [20]. In mouse diabetic conditions, however, oral epithelial cells increasingly express chemokines we have identified by RNAseq, including CXCL1, CXCL2, CXCL3, CXCL10, and CXCL16 [41] but also not CXCL8. Moreover, chemokines such as CXCL8 [42], CXCL10 [43], CXCL12 [44], and CCL5 [45] accumulate in the crevicular fluid of periodontitis patients and their source might be attributed to fibroblasts at least for CXCL2, CXCL3, CXCL8, CXCL9, CXCL10, CXCL12, and CXCL16 [20]. Thus, our HSC2-based bioassay only partially reflects the changes in chemokine expression in a diseased periodontium. We can thus only speculate that EMD may exert part of its activity by decreasing CXCL1, lowering the influx of neutrophils, and CCL20 reducing lymphocytes and antigen-presenting cells in the oral epithelium.

We may further highlight CXCL1 because it is involved in the crosstalk of periodontitis and Crohn’s disease [46]. There is also a link to diabetes as insulin signaling lowers LPS-induced CXCL1 expression in gingival fibroblasts [47], and short-chain fatty acid can lower cytokine-induced expression of CXCL1 and CXCL2 in HSC2 cells and gingival fibroblasts [48]. Higher levels of CXCL1 and other chemokines were found in human and rat’s gingiva from sites of periodontitis as compared with healthy sites [49], and in vitro, biofilms can provoke CXCL1, CXCL3, and CXCL8 expression in epithelial cells [50]. In healthy tissue, however, oral commensal bacteria are associated with the selective expression of CXCL2 but not CXCL1 [51], suggesting CXCL1 is a lead chemokine in oral epithelial cells for periodontitis. CCL20 is also among the chemokines being increased in experimentally induced gingivitis that might become a target of EMD therapy [52]. Support for this assumption would require an epithelial-specific K14-CXCL1 or other chemokine‘s conditional knockout model to prove that a certain chemokine expressed by oral epithelial cells is driving periodontists [53,54].

Our in vitro study has limitations as in vivo cytokines are presumably not the only trigger for oral epithelial cells to become sources of chemokines. For instance, in immortalized human oral keratinocytes, LPS from p. gingivalis caused a robust increase of CXCL1, CXCL8, and CCL20 [55], and in OKF6/TERT-2 oral keratinocytes, LPS increased IL6 [56]. In our in vitro setting, however, LPS at 100 ng/mL from *E. coli* [57] and 1 µg/mL from *P. gingivalis* [48] failed to considerably change chemokines in HSC2 cells, comparable to findings observed by others with the same cell line [58]. This lack of LPS response might be explained by the fact that HSC2 cells, similar to other oral epithelial cells, do not express relevant CD14 required for the LPS-induced signaling via TLR4 [59,60]. However, IFN-γ can upregulate the CD14-TLR signaling machinery and make oral epithelial cells become susceptive to LPS [60,61]. Taken together, oral epithelial cells are no classical targets for LPS; otherwise, the LPS activity of saliva would provoke a chronic inflammation of the oral mucosa [62,63]. Thus, future research might focus on how EMD modulates IFN-γ signaling in HSC2 and other oral epithelial cells, considering the cross-talk of the two signaling pathways. For instance, IFN-γ drives Smad7 expression as a potent inhibitor of canonical TGF-β signaling [64]. It can thus be hypothesized that IFN-γ makes the oral epithelial cell more sensitive to TLR4 signaling and even lowers the capacity of EMD to reduce chemokine expression; however, recent reviews do not support a correlation between IFNγ levels and periodontitis [65]. We can thus propose that the LPS-related anti-inflammatory activity of EMD is presumably more related to macrophages [32,33] than to epithelial cells in vitro. 

Apart from chemokines, our RNAseq has identified a larger spectrum of inflammation-related genes increasingly expressed in HSC2 cells in response to TNFα but not LPS, including the cytokines IL32 [66], IL1β, IL1α–also the IFN-λ1, IFN-λ2, IFN-λ3 (also known as IL29, IL28A, and IL28B, respectively [67,68,69,70,71], as well as interferon-induced GTP-binding protein MX1 and MX2 [72], and the acute-phase protein serum amyloid A1/2 (SAA1 and SAA2) [73]. 

Our rather simple RT-PCR-based approach, however, is not ideal for detecting the impact of EMD on changing the entire signature of TNFα-induced HSC2 cells. Here, an extension of the RNAseq approach towards including EMD and the TGF-β receptor type I kinase-inhibitor SB431542 would help to understand to which extent the TGF-β activity causes the anti-inflammatory activity of EMD in our bioassay proposed, similar to what we have reported for fibroblasts [36]. We have also identified genes being increasingly expressed by both sources of LPS, such as UNC5CL, an inducer of a pro-inflammatory signaling cascade [74]; PTGER4, a prostaglandin E2 receptor expressed in oral squamous cell carcinomas [75] including HSC3 [76]; killin, a p53-regulated nuclear inhibitor of DNA synthesis [77]; and PPP1R3F, the protein phosphatase 1 regulatory subunit 3F. Confirming and interpreting these RNAseq outcomes was not the focus of the present research. Future RNAseq research might also answer if IFN-γ can upregulate the CD14-TLR signaling in HSC2 cells and identify other pathogen-associated molecular patterns than LPS recognized by the large family of toll-like receptors and other pattern recognition receptors to refine the bioassay we have established and continue with the testing EMD and other potential therapies such as platelet-rich fibrin [78,79] in a controlled in vitro setting. 

## 4. Materials and Methods

### 4.1. HSC2 Cells and Primary Oral Epithelial Cells

The oral squamous cell carcinoma cell line HSC2, originally obtained from the Health Science Research Resources Bank (Sennan, Japan) and cultivated in growth Dulbecco’s modified Eagle’s medium (DMEM, Sigma-Aldrich, St. Louis, MO, USA), 10% fetal calf serum (Bio&Sell GmbH, Nuremberg, Germany), and 1% antibiotic-antimycotic (Sigma Aldrich, St.Louis, MO, USA). HSC2 cells were seeded at a concentration of 2.5 × 10^5^ cells/cm^2^ onto culture dishes one day prior to stimulation. Primary oral epithelial cells were taken from the epithelial layer of human gingiva harvested from the extracted third molars of patients who had given informed and written consent. The Ethics Committee of the Medical University of Vienna (EK NR 631/2007) approved the protocol. Primary cells were cultivated in a keratinocyte growth medium (PromoCell, Heidelberg, Germany) and seeded at a concentration of 4.0 × 10^5^ cells/cm^2^ onto culture dishes one day prior to stimulation. In the basic setting, HSC2 and primary epithelial cells were treated overnight with 10 ng/mL TNFα and 10 ng/mL IL-1β (both ProSpec, Ness-Ziona, Israel) with and without 300 µg/mL Emdogain^®^ (EMD; Straumann Group, Basel, Switzerland) or with serum-free medium alone at 37 °C, 5% CO_2_, and 95% humidity before analysis.

### 4.2. RNAseq

RNA quality was evaluated using the Agilent 2100 Bioanalyzer (Agilent Technologies, Santa Clara, CA, USA). Sequencing libraries were prepared at the Core Facility Genomics, Medical University of Vienna, using the NEBNext Poly (A) mRNA Magnetic Isolation Module and the NEBNext Ultra™ II Directional RNA Library Prep Kit for Illumina according to the manufacturer’s protocols (New England Biolabs, Ipswich, MA, USA). Libraries were QC-checked on a Bioanalyzer 2100 (Agilent Technologie, Santa Clara, CA, USA) using a High Sensitivity DNA Kit for correct insert size and quantified using Qubit dsDNA HS Assay (Invitrogen, Thermo Fischer, Waltham, MA, USA). Pooled libraries were sequenced on a NextSeq500 instrument (Illumina, San Diego, CA, USA) in 1 × 75 bp single-end sequencing mode. Approximately 25 million reads were generated per sample. Reads in fastq format were aligned to the human reference genome version GRCh38 (www.ncbi.nlm.nih.gov/grc/human) with Gencode 29 annotations (www.gencodegenes.org/human/release_29.html) using STAR aligner 55 version 2.6.1a in 2-pass mode. Reads per gene were counted by STAR and gene expression was calculated. 

### 4.3. Real-Time Polymerase Chain Reaction (RT-PCR) and Immunoassay

Total RNA was isolated with the ExtractMe total RNA kit (Blirt S.A., Gdańsk, Poland), followed by reverse transcription and polymerase chain reaction (LabQ, Labconsulting, Vienna, Austria) on a CFX Connect™ Real-Time PCR Detection System (Bio-Rad Laboratories, Hercules, CA, USA). The mRNA levels were calculated by normalizing to the housekeeping gene GAPDH using the ΔΔCt method. Primer sequences are in Table 1. For the immunoassays, the human CXCL8 kit (DY208-R&D Systems, Minneapolis, MN, USA) was used. Immunoassay were used according to the manufacturer’s instructions. 

### 4.4. Immunofluorescence Analysis 

Immunofluorescence analysis was performed in HSC2 cells plated on Millicell^®^ EZ slides (Merck KGaA, Darmstadt, Germany). Cells were stimulated with 300 µg/mL EMD or 10 ng/mL TGF-β1, with and without SB431542 (Calbiochem, Merck Millipore, Burlington, MA, USA). Cells were fixed with 4% paraformaldehyde, blocked with 1% bovine serum albumin, and permeabilized with 0.3% Triton X-100 (all Sigma-Aldrich). Smad2/3 (D7G7 XP^®^, Cell Signaling Technology, Danvers, MA, USA) was used overnight at 4 °C. Detection was performed with an Alexa 488 secondary antibody (CS-4412, Cell Signaling Technology). We captured the images on a fluorescence microscope with the DAPI-FITC dual excitation filter block (Echo Revolve Fluorescence Microscope, San Diego, CA, USA).

### 4.5. Western Blot Analysis

HSC2 cells were serum-starved cells and exposed to 300 µg/mL EMD or 10 ng/mL TGF-β1, with and without SB431542, for 30 min. Cell extracts containing SDS buffer with protease and phosphatase inhibitors (cOmplete Ultra Tablets and PhosStop; Roche, Mannheim, Germany) were separated by SDS–PAGE and transferred onto PVDF membranes (Roche Diagnostics, Mannheim, Germany). Membranes were blocked, and the binding of the primary antibodies p-Smad3 (EP823Y, Abcam, Cambridge, UK) and actin (sc-47778, Santa Cruz Biotechnology, Dallas, TX, USA), was detected with the secondary antibody labeled with HRP. After exposure to a substrate (Bio-Rad Laboratories, Inc., Hercules, CA, USA), chemiluminescence signals were visualized with a ChemiDoc imaging system (Bio-Rad Laboratories). In another set of experiments, HSC2 cells exposed to TNFα and IL-1β with and without 300 µg/mL EMD for 30 min were subjected to staining with phospho-NFκB-p65, NFκB-p65 (both Cell Signaling Technology; #7074, #4511), phospho-p38 and p38 (both Santa Cruz Biotechnology; #4511, #535). 

### 4.6. Statistical Analysis and Acronyms

All experiments were performed at least four times. Statistical analyses were performed with paired t-tests or repeated measures one-way ANOVA, with the Geisser–Greenhouse correction tests whenever appropriate. Analyses were performed using Prism v.9 (GraphPad Software; San Diego, CA, USA). Significance was set at *p* < 0.05. The acronyms are listed in Supplementary Table S3.

## 5. Conclusions

Our in vitro approach is based on an unproven assumption that the increased chemokine expression by oral epithelial cells in vivo is a pathological mechanism driving periodontitis. Here, we show data supporting the concept that EMD by activating canonical TGF-β signaling in oral epithelial cells might exert a beneficial effect by normalizing the pathologically increased expression of the chemokines and thereby lower the influx of neutrophils and other cells of the innate immune system driving tissue destruction. Today, this concept remains at the level of a hypothesis but is supported by the data reported here.

## Figures and Tables

**Table 1:** The primer sequences.

Genes	Forward Sequence	Reverse Sequence
CXCL1 [80]	TCCTGCATCCCCCATAGTTA	CTTCAGGAACAGCCACCAGT
CXCL2 [81]	CCCATGGTTAAGAAAATCATCG	CTTCAGGAACAGCCACCAAT
CXCL8 [82]	AACTTCTCCACAACCCTCTG	TTGGCAGCCTTCCTGATTTC
CCL20 [83]	GCTGCTTTGATGTCAGTGCT	GCAGTCAAAGTTGCTTGCTG
GAPDH	AAGCCACATCGCTCAGACAC	GCCCAATACGACCAAATCC

## Data Availability

The original contributions presented in the study are included in the article. Further inquiries can be directed to the corresponding author.

## Supplementary Materials

The following supporting information can be downloaded at: https://www.mdpi.com/article/10.3390/ijms241813991/s1.
