# Supplementary material for: Enamel Matrix Derivative Suppresses Chemokine Expression in Oral Epithelial Cells
Source: Int J Mol Sci. 2023 Sep 12;24(18):13991. doi: 10.3390/ijms241813991 (PMC10530986; doi:10.3390/ijms241813991)
Supplement: Supplementary file 1 [file ijms-24-13991-s001.zip › ijms-2599642-supplementary.pdf]

Supplement Files

Supplement Table S1: RNAseq. HSC2 cells were incubated with LPS *E.coli*, LPS *P.gingivalis,* and TNFα for 24 h. RNA was subjected to RNAseq analysis. Chemokine genes are shown by the expression counts.

Genes	wo	LPS *E.coli*	LPS *P. gingivalis*	TNFα
CXCL1	12	6	16	262
CXCL2	0	1	1	5
CXCL3	2	1	0	22
CXCL8	0	2	3	117
CXCL9	0	0	0	29
CXCL10	0	1	0	996
CXCL11	0	0	0	214
CXCL16	59	98	74	546
CCL5	6	6	9	372
CCL20	0	3	2	47

Supplement Table S2: Gene expression of CXCL8 in HSC2 cells under TNFα and IL-1β stimulation. 10 ng/mL TGF-β and 300 μg/mL EMD reduced expression and 10 μM SB431542 reversed the reduced effect provoked by EMD. Data shows the average and standard deviation of independent experiments.

IL1β+ TNFα	IL1β+ TNFα+TGF-β	IL1β+ TNFα+TGF-β+SB	IL1β+ TNFα+EMD	IL1β+TNFα+EMD+SB
404.1 ± 62.1	155.5 ± 25.9	290.6 ± 38.8	76.3 ± 19.9	250.9 ± 3.6

Supplement Table S3: List of Abbreviations and Acronyms

Abbreviation	Definition
EDM	Enamel matrix derivative
LPS	Lipopolysaccharide
E. coli	Escherichia coli
P. gingivalis	Porphyromonas gingivalis
TNFα	Tumor necrosis factor-alpha
CXCL1	C-X-C motif chemokine ligand 1
CCL5	CC-chemokine ligand 5
IL-1β	Interleukin-1β
NFκB	Nuclear factor kappa B
TGF-β	Transforming growth factor-β
ANOVA	Analysis of variance
RM one-way ANOVA	One-way repeated measures analysis of variance
RNAseq	RNA Sequencing
CD14	cluster of differentiation 14
TLR	toll-like receptor
IFN-λ1	Interferon lambda-1
MX2	MX dynamin like GTPase 2
PPP1R3F	Protein phosphatase 1 regulatory subunit 3F

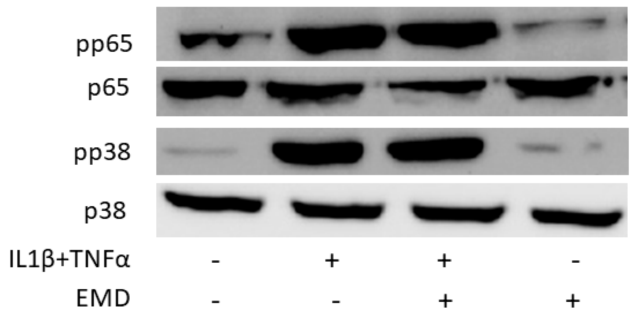

Supplement Figure S1: EMD cannot decrease the p65 and p38 phosphorylation in HSC2 cells.

HSC2 cells were exposed to 300 μg/mL EMD with and without 10 ng/mL IL1β and TNFα aiming to induce the phosphorylation of p65and p38., Western blot analysis shows the chemiluminescence signals obtained with the phosphor-p65 and p65 and phosphor-p38 and p38 antibodies
